# Quantitative ^177^Lu SPECT/CT imaging for personalized dosimetry using a ring-shaped CZT-based camera

**DOI:** 10.1186/s40658-023-00586-z

**Published:** 2023-10-18

**Authors:** Rachele Danieli, Martina Stella, Julian Leube, Johannes Tran-Gia, Clementine Marin, Carlos F. Uribe, Bruno Vanderlinden, Nick Reynaert, Patrick Flamen, Hugo Levillain

**Affiliations:** 1 Department of Medical Physics, Institut Jules Bordet, Hôpital Universitaire de Bruxelles (H.U.B), Université Libre de Bruxelles (ULB), Brussels, Belgium; 2 Radiophysics and MRI Physics Laboratory, Université Libre de Bruxelles (ULB), Brussels, Belgium; 3 GE HealthCare, Diegem, Belgium; 4 Department of Nuclear Medicine, University Hospital Würzburg, Würzburg, Germany; 5 Functional Imaging, BC Cancer, Vancouver, BC Canada; 6 Department of Nuclear Medicine, Institut Jules Bordet, Hôpital Universitaire de Bruxelles (H.U.B), Université Libre de Bruxelles (ULB), Brussels, Belgium

**Keywords:** SPECT/CT, Calibration, Image reconstruction, Block sequential regularized expectation maximization (BSREM), Quantitative Lu-177 SPECT, Dosimetry, CZT digital detectors

## Abstract

**Background:**

Dosimetry after radiopharmaceutical therapy with ^177^Lu (^177^Lu-RPT) relies on quantitative SPECT/CT imaging, for which suitable reconstruction protocols are required. In this study, we characterized for the first time the quantitative performance of a ring-shaped CZT-based camera using two different reconstruction algorithms: an ordered subset expectation maximization (OSEM) and a block sequential regularized expectation maximization (BSREM) combined with noise reduction regularization. This study lays the foundations for the definition of a reconstruction protocol enabling accurate dosimetry for patients treated with ^177^Lu-RPT.

**Methods:**

A series of ^177^Lu-filled phantoms were acquired on a StarGuide™ (GE HealthCare), with energy and scatter windows centred at 208 (± 6%) keV and 185 (± 5%) keV, respectively. Images were reconstructed with the manufacturer implementations of OSEM (GE-OSEM) and BSREM (Q.Clear) algorithms, and various combinations of iterations and subsets. Additionally, the manufacturer-recommended Q.Clear-based reconstruction protocol was evaluated. Quantification accuracy, measured as the difference between the SPECT-based and the radionuclide calibrator-based activity, and noise were evaluated in a large cylinder. Recovery coefficients (RCs) and spatial resolution were assessed in a NEMA IEC phantom with sphere inserts. The reconstruction protocols considered suitable for clinical applications were tested on a cohort of patients treated with [^177^Lu]Lu-PSMA-I&T.

**Results:**

The accuracy of the activity from the cylinder, although affected by septal penetration, was < 10% for all reconstructions. Both algorithms featured improved spatial resolution and higher RCs with increasing updates at the cost of noise build-up, but Q.Clear outperformed GE-OSEM in reducing noise accumulation. When the reconstruction parameters were carefully selected, similar values for noise (~0.15), spatial resolution (~1 cm) and RCs were found, irrespective of the reconstruction algorithm. Analogue results were found in patients.

**Conclusions:**

Accurate activity quantification is possible when imaging ^177^Lu with StarGuide™. However, the impact of septal penetration requires further investigations. GE-OSEM is a valid alternative to the recommended Q.Clear reconstruction algorithm, featuring comparable performances assessed on phantoms and patients.

**Supplementary Information:**

The online version contains supplementary material available at 10.1186/s40658-023-00586-z.

## Background

Radiopharmaceutical therapy (RPT) with lutetium-177 (^177^Lu) has gained popularity due to its proven efficacy in the treatment of inoperable somatostatin receptor-positive gastroenteropancreatic neuroendocrine tumours [1] and, more recently, metastatic castration-resistant prostate cancer [2].

Current protocols adopt a "one-size-fits-all" approach, where all patients are administered the same amount of activity, typically around 7.4 GBq. However, these protocols fail to account for the individual variations in physiological and anatomical characteristics among patients, resulting in suboptimal treatment outcomes.

Personalized dosimetry is a promising tool that can enable treatment optimization [3]. There is growing evidence that the absorbed dose to tumours and organs at risk can be directly correlated to the treatment efficacy and toxicity, respectively [4–7]. By utilizing dosimetry, it becomes possible to determine the ideal administered activity tailored to each patient's unique characteristics, thereby leading to optimized treatment outcomes [8].

Personalized dosimetry requires quantitative imaging techniques to assess the radiopharmaceutical biodistribution and biokinetics in the patient. During its decay into an excited state of Hafnium-177 (^177^Hf), ^177^Lu releases beta particles that are suitable for therapeutic applications. Additionally, the resulting ^177^Hf emits gammas at 113 keV and 208 keV, which are well suited for quantitative SPECT/CT imaging. When imaging these gammas, however, conventional SPECT systems based on scintillators face limitations. The emission probabilities of these gammas that are relatively low (6.2% for 113 keV and 10.4% for 208 keV)- and medium-energy collimators are recommended [9]. Consequently, the acquisition times for whole-body tomographic imaging using these systems are typically long, ranging from 20 to 30 min per bed position [10]. This makes whole-body SPECT/CT imaging impractical for routine clinical use.

Recently, commercial general-purpose SPECT systems based on semiconductors, such as cadmium–zinc–telluride (CZT), have been introduced. These systems provide superior sensitivity, higher energy resolution and better image contrast than standard systems [11]. As a result, they can lead to shorter acquisition times of quantitative post-therapeutic ^177^Lu imaging, possibly enabling routine clinical use of quantitative whole-body SPECT/CT imaging.

Fast whole-body (12 min—vertex to mid-thighs) SPECT/CT after ^177^Lu-RPT with StarGuide™ (GE HealthCare)—a 360° ring-shaped CZT-based SPECT/CT system—has already proved to be feasible and to provide a detection/targeting rate comparable to a conventional system [12]. However, to the best of our knowledge, no studies on the quantitative performance of StarGuide™ for dosimetry after ^177^Lu-RPT have been published yet. Prior to implementing dosimetry in the clinic, phantom-based studies or Monte Carlo phantom simulations with a known ground truth are necessary to define suitable imaging protocols and to assess their accuracy with respect to the activity quantification [13]. This is particularly important when new technologies, both hardware (e.g. CZT-based detectors) and software (e.g. reconstruction algorithms), are introduced, as in the case of StarGuide™. The ultimate goal of these preliminary studies is to enable, through accurate and precise activity quantification, the assessment of dose–effect correlations based on which personalized treatment planning can be implemented.

The aim of this study, encompassing both phantom and patient data, is to characterize for the first time the quantitative performance of StarGuide™ for quantitative ^177^Lu SPECT/CT imaging in relation to the reconstruction protocol. With this study, we want to lay the foundations for the definition of an optimal reconstruction protocol enabling accurate dosimetry for patients treated with ^177^Lu-RPT undergoing post-therapeutic imaging with StarGuide™.

## Methods

A series of ^177^Lu-filled phantoms measurements were taken to assess the quantification accuracy, the noise build-up and the spatial resolution for two different reconstruction algorithms: i) a conventional ordered subset expectation maximization (OSEM) and ii) a block sequential regularized expectation maximization (BSREM) [14]. Based on the results of this phantom study, the reconstruction protocols considered suitable for clinical routine were tested on a cohort of 10 patients treated with [^177^Lu]Lu-PSMA-I&T that underwent post-therapeutic imaging.

All phantom and patient acquisitions were performed with StarGuide™.

### StarGuide™ SPECT/CT system

StarGuide™ is a hybrid system consisting of a CT, Optima CT540, and a ring-shaped gantry with twelve detectors. Each detector, consisting of seven CZT modules of 16 × 16 2.46 mm pixel size and 7.25 mm thickness, is independent of the others and is capable of both radial (in/out) and rotational (sweep) motion. In addition, all the detectors can simultaneously rotate with the gantry. The camera is equipped with a fixed dual-channel collimator, which enables a balance between sensitivity and resolution across low- and medium-energy isotopes. Before each acquisition, an infrared-based technology scans the contours of the patient (or phantom) to be acquired, enabling the automatic positioning of the table and detectors as closely as possible to the imaged object, maximizing the resolution.

StarGuide™ is equipped with a dedicated Web-based processing station for image reconstruction, SmartConsole. Besides the manufacturer implementation of the OSEM algorithm, referred to as GE-OSEM, SmartConsole provides a new iterative algorithm for image reconstruction, Q.Clear. Q.Clear is based on a BSREM [14] algorithm and, contrary to OSEM, allows replacing the standard maximum likelihood with a penalized likelihood objective function including a regularization term, in the form of either a relative difference prior (RDP) [15] or a median root prior (MRP) [16]. In case of RDP regularization, which was used in this study, two parameters, called beta and gamma, can be used to fine-tune the regularization. Optionally, the beta parameter can be weighted according to a sensitivity map derived by the CT-based attenuation map by turning on the tool bySens. Both algorithms incorporate non-optional resolution recovery. CT-based attenuation correction and scatter correction can be also applied. The latter is a dual energy window correction that takes into account the “tailing” effect typical of CZT-based detectors [17].

SPECT images obtained with SmartConsole are expressed in units of counts per voxel.

### Phantom study

#### Phantom preparation

Three phantoms were prepared: i) a Flangeless Deluxe Jaszczak Phantom™ (“Jaszczak”, filling volume: 5805 mL, diameter: 20.9 cm, height: 18.6 cm), ii) a cylindrical phantom without inserts (“cylinder phantom”, filling volume: 5440 mL, diameter: 20 cm, height: 18 cm) and iii) a NEMA IEC phantom with 6 spherical inserts (“NEMA phantom”, diameters: 10, 13, 17, 22, 28, 37 mm). The Jaszczak and the cylinder phantom were filled with 541 MBq and 700 MBq of labelled ^177^Lu, respectively, to generate activity concentrations typically observed in images of patients treated with ^177^Lu-RPT. To mimic the activity concentrations found within tumours, the spherical inserts of the NEMA phantom were filled with 1.6 MBq/mL of ^177^Lu. After a first acquisition without activity in the background, 1.5 GBq of ^177^Lu was added to the background of the NEMA phantom to obtain a sphere-to-background ratio of about 10:1.

All the phantoms presented in this study were prepared with the addition of an excess of ethylenediaminetetraacetic acid (EDTA) to prevent ^177^Lu from sticking to the phantom walls. Nominal activities were determined using a radionuclide calibrator equipped with a VIK-202 ionization chamber (Comecer SpA). The calibration factor for ^177^Lu was provided by the manufacturer. Injected activities were computed taking into account the residual activity measured in the syringe used to fill the phantoms.

#### Image acquisition and reconstruction

Projections were acquired in continuous sweep mode with steps of 2° over 15 min. The photopeak and scatter energy windows, defined as suggested by the manufacturer, were centred at 208(± 6%) keV and 185(± 5%) keV, respectively. After the SPECT acquisition, a low-dose CT was acquired for attenuation correction (120 kV, 512 × 512 × 112 matrix, 1.0 × 1.0 × 2.5 mm^3^ resolution).

Data were reconstructed using SmartConsole (GE HealthCare, version 1.0.10). The impact of the regularization on the quantitative performance was studied by reconstructing images using Q.Clear without regularization, hereafter named Q.Clear, and with an RDP regularization (gamma = 1, beta = 0.005, bySens OFF), hereafter named Q.ClearRDP. Hence, each phantom was reconstructed with three algorithms: i) GE-OSEM, ii) Q.Clear and iii) Q.ClearRDP. All reconstructions included CT-based attenuation correction, scatter correction and resolution recovery. No post-filtering was applied, as suggested by the EANM dosimetry committee recommendations for dosimetry of ^177^Lu-labelled somatostatin receptor- and PSMA-targeting ligands [9]. To study the influence of the number of updates (i.e. the number of iterations times the number of subsets) for each algorithm (GE-OSEM, Q.Clear and Q.ClearRPD), images were reconstructed with 1 subset and several numbers of iterations between 12 and 576 (12i1s stands for 12 iterations and 1 subset). In addition, the influence of the number of subsets was investigated by fixing the number of updates and varying the partitioning into iterations and subsets. All data were reconstructed using the intrinsic voxel size of the CZT modules (2.46 mm). The matrix size was automatically determined by the system.

For comparison, all images were also reconstructed according to the protocol suggested by the manufacturer, hereafter named factory protocol, corresponding to Q.Clear 20i10s with RDP regularization (gamma = 1, beta = 0.08, bySens ON).

#### Image analysis

Image analysis was performed using an in-house Python code (Python version 3.10.0).

#### Image calibration factor

The Jaszczak phantom was used to determine an image calibration factor (ICF) to convert reconstructed counts into activity concentration. For each reconstruction, the ICF was obtained as:

$$\mathrm{ICF}=\frac{C}{{T}_{\mathrm{acq}}\times {A}_{\mathrm{prep}}\times \mathrm{exp}\left(\frac{-\mathrm{ln}(2)}{{T}_{1/2}}\times \Delta t\right)}\left[\frac{\mathrm{cps}}{\mathrm{MBq}}\right]$$

where $C$ is the total number of counts inside a large volume of interest (VOI) drawn around the reconstructed phantom (diameter: 23 cm, height: 22 cm), ${T}_{\mathrm{acq}}$ is the acquisition duration [s], ${A}_{\mathrm{prep}}$ is the activity at the time of phantom preparation [MBq], $\Delta t$ is the time between phantom preparation and acquisition [s] and ${T}_{1/2}$ is the physical half-life of ^177^Lu [s]. The uncertainty associated with the ICF was calculated using the law of propagation of uncertainty. Assuming no uncertainty on the time measurements, the uncertainty of the ICF can be written as:

$$u(\mathrm{ICF})=\mathrm{ICF}\times \sqrt{{\left(\frac{u\left(C\right)}{C}\right)}^{2}+{\left(\frac{u\left({A}_{\mathrm{prep}}\right)}{{A}_{\mathrm{prep}}}\right)}^{2}}$$

The standard uncertainty of the counts was computed as the square root of the number of reconstructed counts $C: u\left(C\right)=\sqrt{C}$ [18]. The uncertainty associated with the activity (${A}_{\mathrm{prep}}$) was assumed to be 2%, based on the mean deviation between the activity measured by the ^177^Lu provider (ITM Isotope Technologies Munich SE) and the on-site measurement over 190 vials received from July 2021 to November 2022 [19].

#### Quantification accuracy

The cylinder phantom was used to assess the quantification accuracy. A large cylindrical VOI (“VOI_outside”, diameter: 22.0 cm, height: 20.0 cm, volume: 7.9 L) and a small cylindrical VOI (“VOI_inside”, diameter: 14.0 cm, height: 12.6 cm, volume: 1.9 L) were drawn around and inside the cylinder phantom, respectively. For each reconstruction, the activity in VOI_outside and the activity concentration in VOI_inside were calculated by applying the ICF corresponding to the same reconstruction protocol. The accuracy in the quantification of the activity (or activity concentration) was computed as the percentage difference between the SPECT-based (${A}_{\mathrm{meas}}$) and the radionuclide calibrator-based activity (or activity concentration, ${A}_{\mathrm{prep}}$):

$$\mathrm{Error}=({A}_{\mathrm{meas}}-{A}_{\mathrm{prep}})/{A}_{\mathrm{prep}}$$

#### Image quality

The cylinder and the NEMA phantom were used to determine the noise and the spatial resolution, respectively, as a function of the reconstruction parameters. The noise was computed as coefficient of variation $\mathrm{CV}$ (i.e. the ratio between the standard deviation and the mean) of counts in VOI_inside. The spatial resolution was calculated using a matched-filter resolution analysis based on the NEMA phantom measurement [20]. The cross section through the centre of the cylinder phantom was visually analysed to assess the noise build-up as a function of the number of updates.

#### Recovery coefficient

To assess the relationship between object size and measurement accuracy, the NEMA phantom was used. Firstly, the six spheres were manually delineated on each CT using MIM (MIM Software Inc., version 7.3.1). Then, each reconstructed SPECT image was resampled to CT resolution using a linear interpolation algorithm. Finally, each set of spherical VOIs was transferred to the corresponding interpolated SPECT image, and for each sphere, a recovery coefficient (RC) was computed as:

$$\mathrm{RC}=\frac{C}{{T}_{\mathrm{acq}}\times \mathrm{ICF}\times V}\times \frac{1}{{c}_{\mathrm{prep}}\times \mathrm{exp}\left(\frac{-\mathrm{ln}(2)}{{T}_{1/2}}\times \Delta t\right)}[\%]$$

where $\mathrm{ICF}$ is the calibration factor for the corresponding reconstruction protocol [cps/MBq], $V$ is the volume of the spherical VOI [mL] and ${c}_{\mathrm{prep}}$ is the activity concentration at the time of preparation [MBq/mL].

To visually assess the recovery of the activity concentration inside the spherical VOIs as a function of the number of updates, the cross section through the centre of the largest sphere was plotted and compared to the ideal profile (a rectangular function).

#### Optimal combination of iterations and subsets

Since no objective criteria are currently available for defining an optimized reconstruction protocol for quantitative ^177^Lu SPECT/CT, the optimal iteration–subset combination for each algorithm was defined as the one yielding a noise level equivalent to that obtained with the factory protocol, used as the reference.

### Patient study

#### Image acquisition and reconstruction

Ten patients treated with [^177^Lu]Lu-PSMA-I&T (mean(± standard deviation) injected activity: 7134(± 248) MBq) who underwent SPECT/CT imaging with StarGuide™ (mean(± standard deviation) time after the injection: 18(± 1) hours) were selected. Depending on patient’s size, 4 to 5 bed positions were acquired. Acquisition parameters were defined as in the phantom experiments, except for the acquisition duration that was equal to 2.5 min for the head and legs bed positions, and 5 min for the torso bed position. Acquired projections were reconstructed using GE-OSEM, Q.Clear and Q.ClearRDP, with a number of updates optimized as explained above, and the factory protocol.

#### Image analysis

Twenty-seven tumours with a volume larger than 5 mL were selected. Tumour delineation was performed based on the factory protocol reconstructions. Firstly, the PERCIST SUV Peak tool in MIM was used to calculate the largest possible mean value in a spherical 1-mL VOI placed within the tumour. Secondly, tumours were delineated using a 30%-isocontour of the mean value previously determined [21]. The resulting mask was then applied to all other reconstructions. Lastly, the activity concentration in each tumour was measured by applying the ICF.

#### Statistical analysis

Values are presented as relative percentage difference compared to the factory protocol. A Wilcoxon matched-pairs signed-rank test (GraphPad Prism, version 7.05) was used to compare the activity concentration measured according to each optimized protocol and the factory protocol. A statistical significance for *P* values less than 0.05 was considered.

## Results

For the sake of clarity, results for a limited number of reconstructions (12i1s, 24i1s, 48i1s, 96i1s, 192i1s, 288i1s, 576i1s, 12i16s) will be presented in the following sections. Unless relevant for comparison, results for the NEMA phantom with hot background will be given in Additional file 1, being considered as less representative of patients treated with [^177^Lu]Lu-PSMA-I&T.

### Image calibration factor

Values of the ICF for GE-OSEM, Q.Clear and Q.ClearRDP reconstructions for different numbers of iterations between 12 and 576 and 1 subset are given in Table 1. The ICF for the factory protocol was found equal to 94.6 cps/MBq.

**Table 1:** Image calibration factors for GE-OSEM, Q.Clear and Q.ClearRDP for different numbers of iterations and 1 subset

Algorithm	Image calibration factor [cps/MBq]						
	12i1s	24i1s	48i1s	96i1s	192i1s	288i1s	576i1s
GE-OSEM	89.5	93.2	93.8	94.0	93.9	93.9	93.8
Q.Clear	87.9	92.6	93.6	93.9	93.9	94.0	94.0
Q.ClearRDP	87.9	92.6	93.7	94.0	94.1	94.2	94.2

Regardless of the reconstruction algorithm, the ICF increased by an average of 6% from 12 to 48 updates. From 48 to 576 updates, no major increase in the ICF was observed, suggesting that convergence was reached. No dependency of the ICF on the reconstruction algorithm (Table 1) nor on the number of subsets for a fixed number of updates (Additional file 1: Table 1) was found. The uncertainty of the ICF, dominated by the uncertainty in the dose calibrator, was found to be equal to 2%, regardless of the reconstruction algorithm and parameters.

### Quantification accuracy

The errors in the activity and activity concentration quantification measured on the cylinder phantom acquired projections reconstructed with GE-OSEM, Q.Clear and Q.ClearRDP are given in Table 2. For the factory protocol, those errors were found equal to − 1.3% and − 8.7%, respectively.

**Table 2:** Percentage error in the quantification of the activity in VOI_outside and of the activity concentration in VOI_inside for the cylinder phantom scan reconstructed with GE-OSEM, Q.Clear and Q.ClearRDP for different numbers of iterations and 1 subset

Algorithm	Metric	12i1s	24i1s	48i1s	96i1s	192i1s	288i1s	576i1s
GE-OSEM	Error VOI_outside [%]	− 1.8	− 1.8	− 1.4	− 1.0	− 1.1	− 1.2	− 1.2
	Error VOI_inside [%]	3.8	− 4.0	− 8.5	− 9.0	− 7.8	− 7.6	− 7.6
Q.Clear	Error VOI_outside [%]	− 1.8	− 1.8	− 1.6	− 1.4	− 1.2	− 1.2	− 0.6
	Error VOI_inside [%]	4.8	− 1.7	− 6.8	− 8.8	− 8.8	− 8.7	− 7.9
Q.ClearRDP	Error VOI_outside [%]	− 1.9	− 1.8	− 1.8	− 1.6	− 1.4	− 1.5	− 0.8
	Error VOI_inside [%]	4.8	− 1.6	− 6.8	− 8.9	− 9.1	− 9.1	− 8.3

The error in the quantification of the activity measured in VOI_outside was below 2% for all investigated reconstructions. For VOI_inside, however, higher discrepancies were found. After more than 48 updates, the activity concentration was underestimated on average by 8%. No dependency of the quantification accuracy on the reconstruction algorithm (Table 2) nor on the number of subsets for a fixed number of updates (Additional file 1: Table 2) was found.

### Image quality

**Fig. 1:**
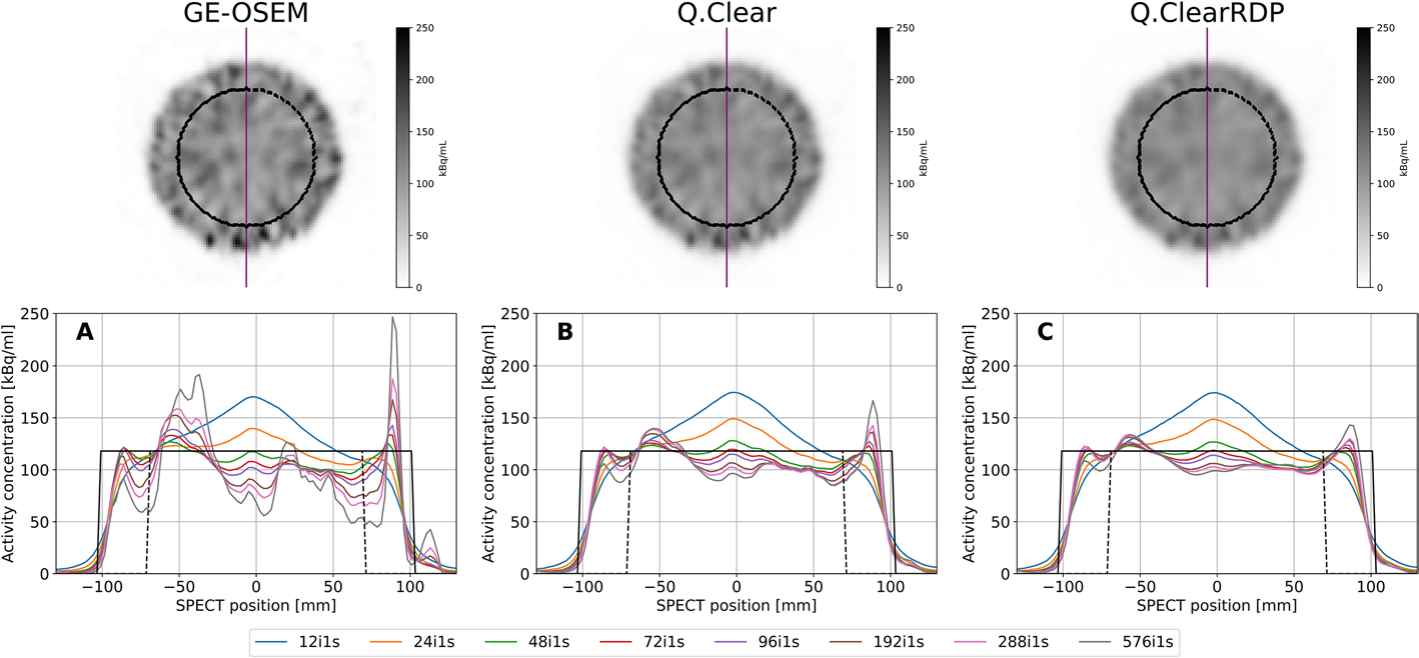
Top panel—axial views of the cylinder phantom reconstructed using GE-OSEM, Q.Clear and Q.ClearRDP (96i1s). The purple line defines the cross section presented in the bottom panel, while the dashed contour corresponds to VOI_inside. Bottom panel—cross section through the cylinder phantom images reconstructed with GE-OSEM (**A**), Q.Clear (**B**) and Q.ClearRDP (**C**) for different numbers of iterations and 1 subset. The black continuous and dashed rectangular functions represent the ideal and the VOI_inside profiles, respectively

**Fig. 2:**
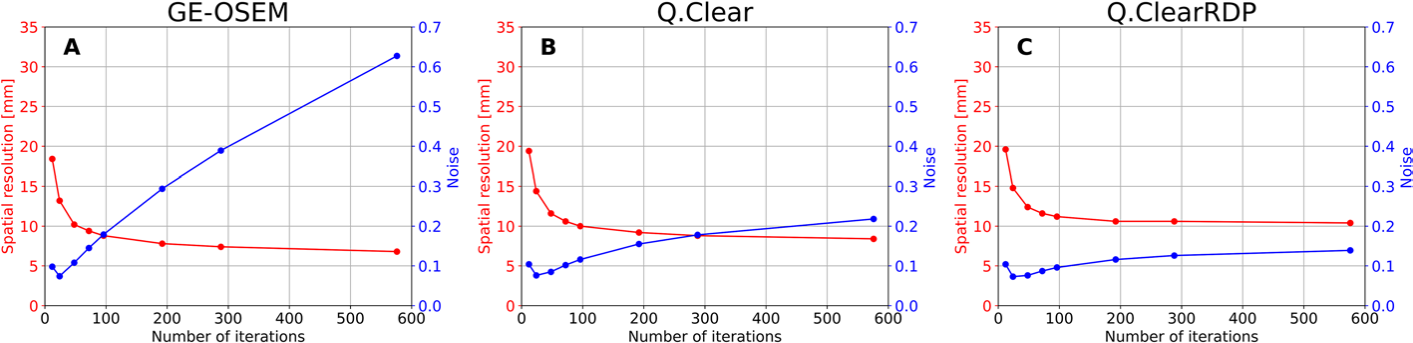
Spatial resolution (red), computed on the NEMA phantom with cold background, and noise (blue) as a function of the number of updates (1 subset) for GE-OSEM (**A**), Q.Clear (**B**) and Q.ClearRDP (**C**). For the factory protocol, spatial resolution and noise were found equal to 9.8 mm and 0.15, respectively

The update dependency of the noise is illustrated in Fig. 1, where the cross sections through the reconstructed cylinder phantom for increasing iterations (1 subset) are shown. Figure 2 shows the noise and the spatial resolution measured on the cylinder and the NEMA phantom with cold background, respectively, as a function of the number of iterations (1 subset) for GE-OSEM, Q.Clear and Q.ClearRDP.

After 12 iterations, all algorithms provided similar noise ($CV$~ 0.10) and spatial resolution (~ 20 mm). For increasing numbers of iterations, however, GE-OSEM presented a faster noise build-up than Q.Clear and Q.ClearRDP ($CV$ of 0.63, 0.22 and 0.14, respectively, after 576 iterations). This is clearly visible in Fig. 2, suggesting that the reconstruction parameters providing an optimal trade-off between noise build-up and resolution improvement are different in the three cases. After 48 iterations, a resolution of 10.2 mm, 11.6 mm and 12.4 mm was determined for GE-OSEM, Q.Clear and Q.ClearRDP, respectively. From 48 to 576 iterations, an improvement down to 6.8 mm, 8.4 mm and 10.4 mm was observed.

Results for the hot background are shown in Additional file 1: Fig. 1. Activity in the background strongly deteriorated the spatial resolution, converging at 9.0 mm, 12.0 mm and 14.4 mm for GE-OSEM, Q.Clear and Q.ClearRDP, respectively, after 576 iterations (1 subset).

When fixing the number of updates, the quality of images reconstructed with Q.Clear and, to a lesser extent, with Q.ClearRDP was found to be dependent on the number of subsets_._ As example, the spatial resolution and the noise corresponding to 192i1s and 12i16s are given in Table 3.

**Table 3:** Spatial resolution and noise for GE-OSEM, Q.Clear and Q.ClearRDP with 192i1s and 12i16s

Reconstruction		Spatial resolution (cold bgr) [mm]	Spatial resolution (hot bgr) [mm]	Noise
GE-OSEM	192i1s	7.8	10.8	0.29
	12i16s	7.8	10.6	0.30
Q.Clear	192i1s	9.2	13.8	0.16
	12i16s	8.0	10.8	0.27
Q.ClearRDP	192i1s	10.6	15.6	0.12
	12i16s	10.4	13.6	0.15

### Recovery coefficients

**Fig. 3:**
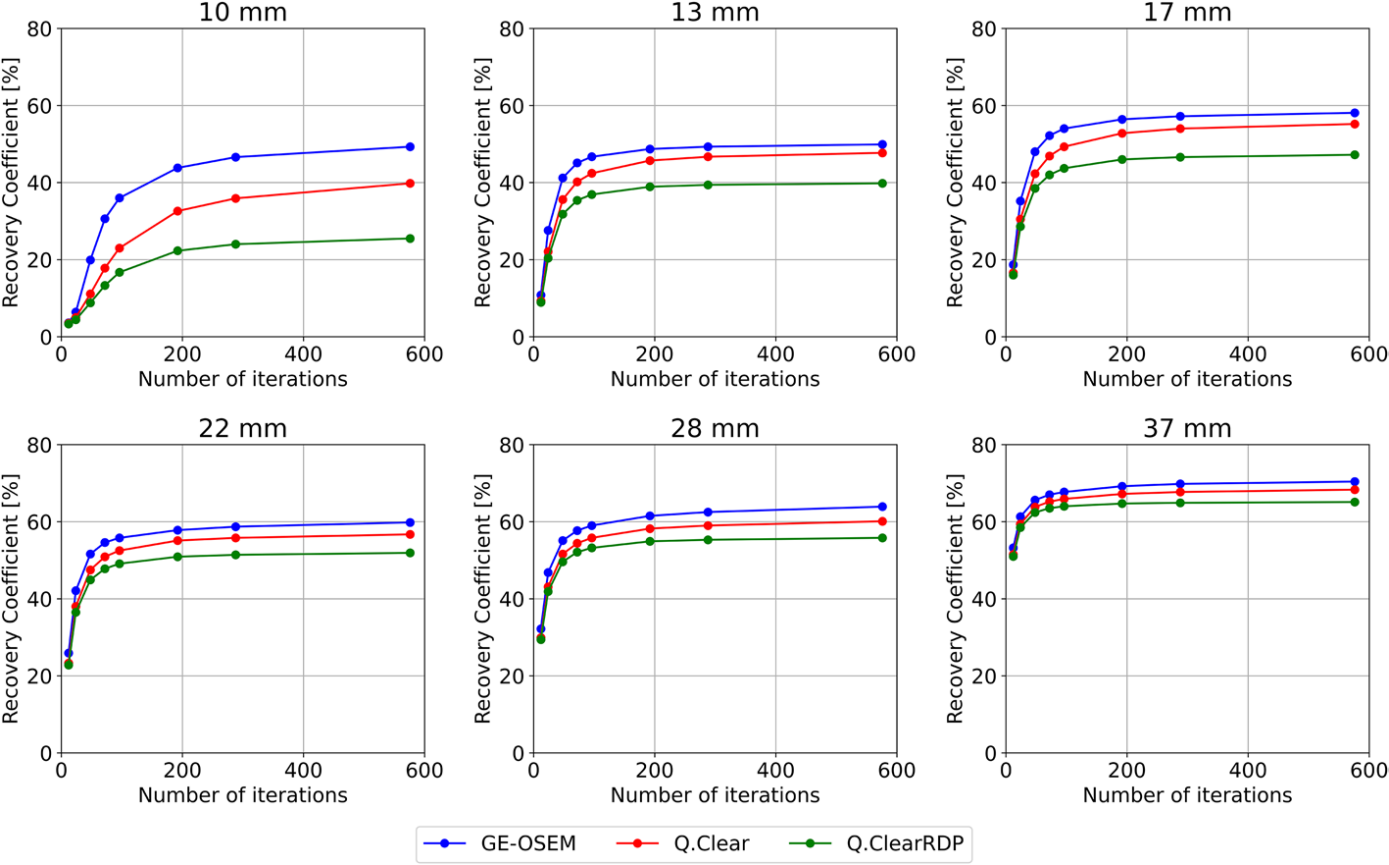
Recovery coefficients for the spherical inserts of the NEMA phantom with cold background as a function of the number of iterations (1 subset) for GE-OSEM, Q.Clear and Q.ClearRDP. For the factory protocol, RCs with cold background were found equal to 26%, 37%, 49%, 54%, 58% and 66% for the 10 mm, 13 mm, 17 mm, 22 mm, 28 mm and 37 mm sphere, respectively

The RCs for the spherical inserts of the NEMA phantom with cold background for GE-OSEM, Q.Clear and Q.ClearRDP are shown as a function of the number of iterations and noise (1 subset) in Fig. 3 and Additional file 1: Fig. 2, respectively.

GE-OSEM reconstructions resulted in higher RCs than Q.Clear and Q.ClearRDP, although differences decreased with increasing sphere volume. After 48 iterations, the RCs for the largest sphere were 66%, 64% and 62% for GE-OSEM, Q.Clear and Q.ClearRDP, respectively. From 48 to 576 iterations, a minor improvement up to 70%, 68% and 65% was observed. A slower rate of convergence was observed for decreasing sphere sizes.

**Fig. 4:**
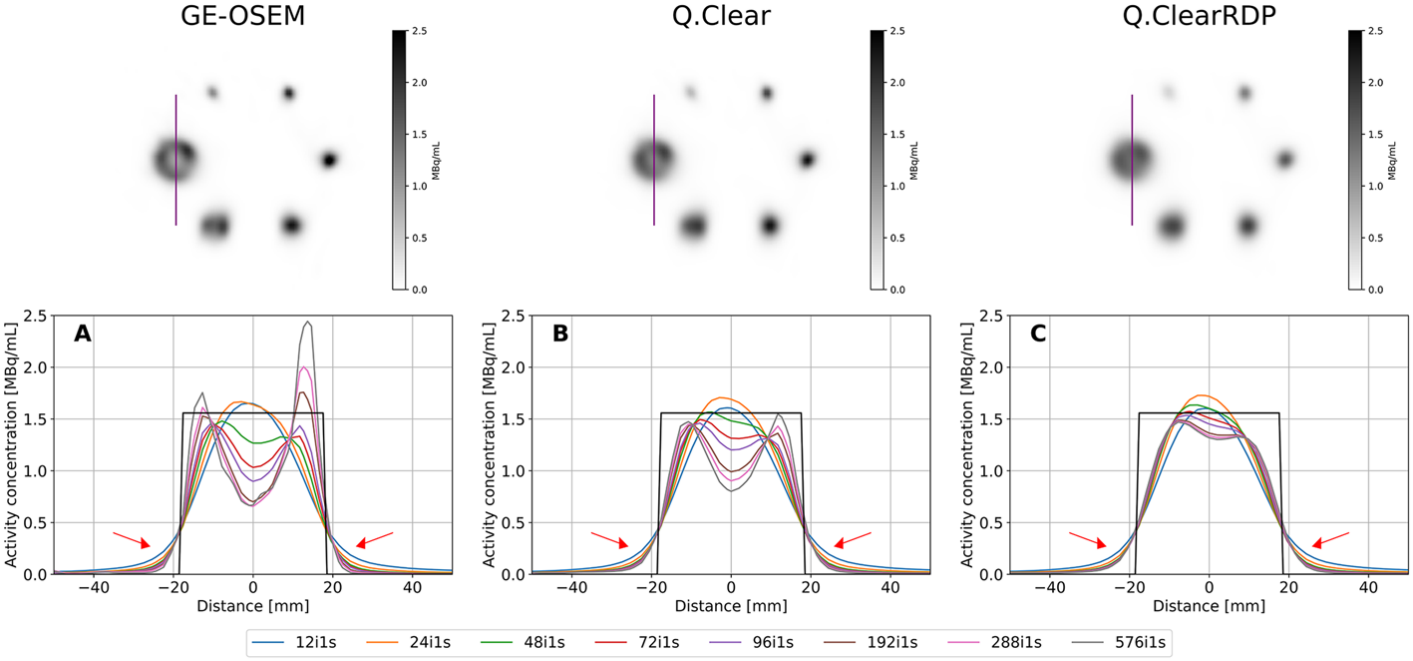
Top panel—axial views of the NEMA phantom with cold background reconstructed using GE-OSEM, Q.Clear and Q.ClearRDP (96i1s). The purple line through the biggest sphere defines the cross section presented in the bottom panel. Bottom panel—cross section through the largest sphere for GE-OSEM (**A**), Q.Clear (**B**) and Q.ClearRDP (**C**) for different numbers of iterations and 1 subset. The black rectangular function represents the ideal profile. The red arrows point at the activity spill-out

Figure 4 shows the cross section through the largest sphere of the NEMA phantom with cold background for different reconstructions. With equal number of iterations, activity spill-out was more evident for the Q.Clear and Q.ClearRDP than for GE-OSEM reconstructions (red arrows in Fig. 4—bottom panel), explaining the lower RCs (Fig. 3). On the contrary, more evident Gibbs-like artefacts (i.e. appearance of a spherical shell) were observed on GE-OSEM than on Q.Clear and Q.ClearRDP reconstructions (Fig. 4).

Results for the NEMA phantom with hot background are shown in Additional file 1: Figs. 3 and 5. Overall, RCs were considerably deteriorated by the presence of activity in the background, especially for the smaller spheres. Visually, the smallest sphere was barely visible. Moreover, all the spherical inserts presented a less sphere-like shape than in the cold background case (Additional file 1: Fig. 4—top panel). Although the activity outside the largest sphere reached the nominal value on both sides, the profile was strongly asymmetric (Additional file 1: Fig. 4—bottom panel). No Gibbs-like artefacts were observed.

When fixing the number of updates, the RCs measured on Q.Clear and Q.ClearRDP reconstructions were found to be dependent on the number of subsets, while no dependency on the number of subsets was found for GE-OSEM. As an example, the RCs measured on GE-OSEM, Q.Clear and Q.ClearRDP reconstructions with 192i1s and 12i16s are given in Table 4.

**Table 4:** Recovery coefficients for GE-OSEM, Q.Clear and Q.ClearRDP with 192i1s and 12i16s measured on the NEMA phantom with cold and hot background

Reconstruction		Recovery coefficient [%] (cold background)						Recovery coefficient [%] (hot background)					
		10 mm	13 mm	17 mm	22 mm	28 mm	37 mm	10 mm	13 mm	17 mm	22 mm	28 mm	37 mm
GE-OSEM	192i1s	44	49	56	58	62	69	22	45	41	53	59	68
	12i16s	45	47	52	58	61	71	22	48	43	53	60	69
Q.Clear	192i1s	33	46	53	55	58	67	17	30	29	41	50	64
	12i16s	44	46	52	57	61	70	22	44	41	52	59	68
Q.ClearRDP	192i1s	22	39	46	51	55	65	15	24	24	35	46	60
	12i16s	28	39	44	51	56	67	17	29	28	41	52	63

### Optimal combination of iterations and subsets

**Fig. 5:**
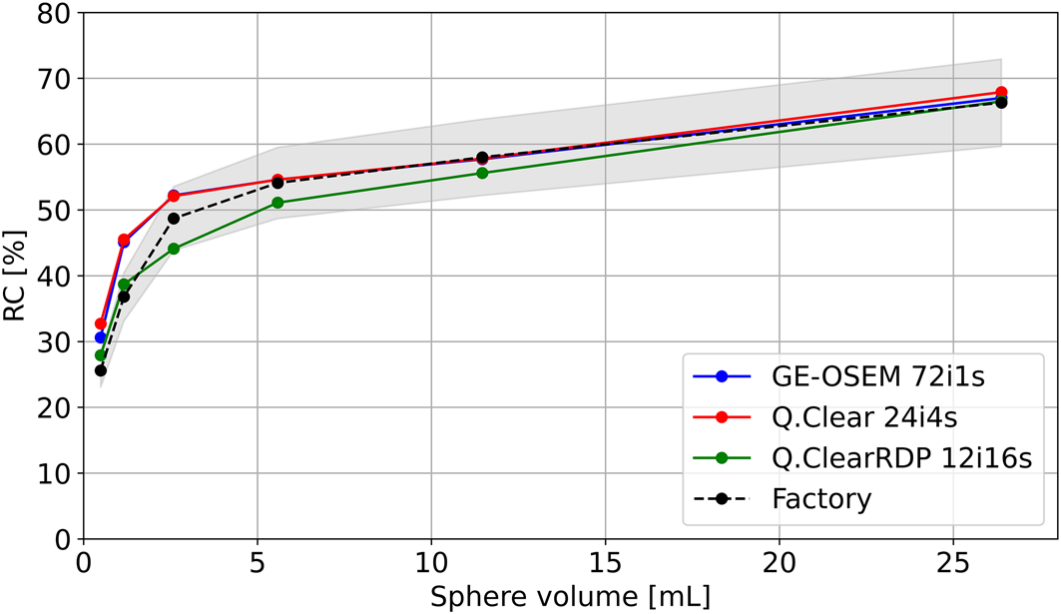
Recovery coefficients for the NEMA phantom filled with cold background reconstructed with GE-OSEM 72i1s, Q.Clear 24i4s and Q.ClearRDP 12i16s and factory protocol as a function of the sphere volume. Shaded regions correspond to ± 10% the RCs computed on the factory protocol reconstruction

**Fig. 6:**
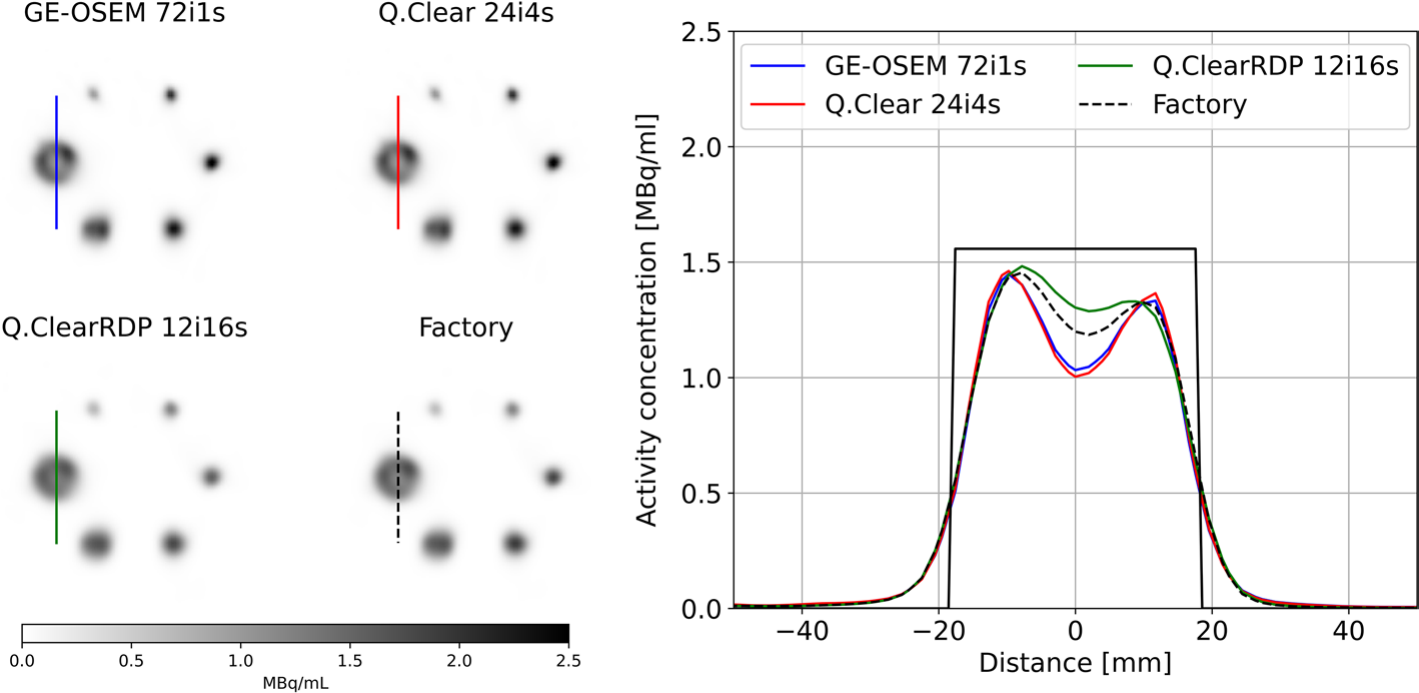
Left panel—axial views of the NEMA phantom with cold background reconstructed using GE-OSEM 72i1s, Q.Clear 24i4s and Q.ClearRDP 12i16s and factory protocol. The line through the largest sphere defines the cross section presented in the right panel. Right panel—cross section through the largest sphere for GE-OSEM 72i1s, Q.Clear 24i4s and Q.ClearRDP 12i16s and factory protocol. The black rectangular function represents the ideal profile

When using the factory protocol, a $CV$ of about 0.15 was measured. Similar levels of noise were found for GE-OSEM 72i1s, Q.Clear 24i4s and Q.ClearRDP 12i16s. The results for the quantification accuracy and spatial resolution for GE-OSEM 72i1s, Q.Clear 24i4s, Q.ClearRDP 12i16s and the factory protocol are listed in Table 5. For the same protocols, the RCs for cold and hot backgrounds are shown in Fig. 5 and Additional file 1: Fig. 6, respectively. Example axial views of the NEMA phantom reconstructions and cross sections through the biggest sphere are given in Fig. 6 and Additional file 1: Fig. 7 for cold and hot backgrounds, respectively.

**Table 5:** Quantitative accuracy and spatial resolution for GE-OSEM 72i1s, Q.Clear 24i4s and Q.ClearRDP 12i16s and factory protocol

Algorithm and parameters	Error VOI_outside [%]	Error VOI_inside [%]	Spatial resolution (cold bgr) [mm]	Spatial resolution (hot bgr) [mm]
GE-OSEM 72i1s	− 1.2	− 8.9	9.4	14.2
Q.Clear 24i4s	− 0.9	− 8.6	9.2	15.6
Q.ClearRDP 12i16s	− 1.1	− 8.1	10.4	13.6
Factory	− 1.3	− 8.7	9.8	13.6

### Patient study

The mean(± standard deviation) lesion volume and activity concentration (measured on the factory protocol reconstructions) were 11.7(± 5.8) mL (range: 5.3–29.5 mL) and 864(± 1022) kBq/mL (range: 204–5648 kBq/mL), respectively.

Descriptive statistics for the relative percentage difference between the activity concentration measured with each of the optimized protocols and the factory protocol are given in Table 6. *P* values resulting from Wilcoxon matched-pairs signed-rank tests are reported.

**Table 6:** Minimum, first quartile (Q1), median, third quartile (Q3) and maximum relative percentage difference between the activity concentrations measured with each of the optimized protocols and the factory protocol

	Activity concentration					
	Minimum (%)	Q1 (%)	Median (%)	Q3 (%)	Maximum (%)	*P*
GE-OSEM 72i1s	− 19	− 5	− 2	1	3	0.0965
Q.Clear 24i4s	− 15	− 3	0	3	4	0.6318
Q.ClearRDP 12i16s	− 40	− 12	− 8	− 3	1	< 0.0001

*P* values resulting from Wilcoxon matched-pairs signed-rank tests are also reported

**Fig. 7:**
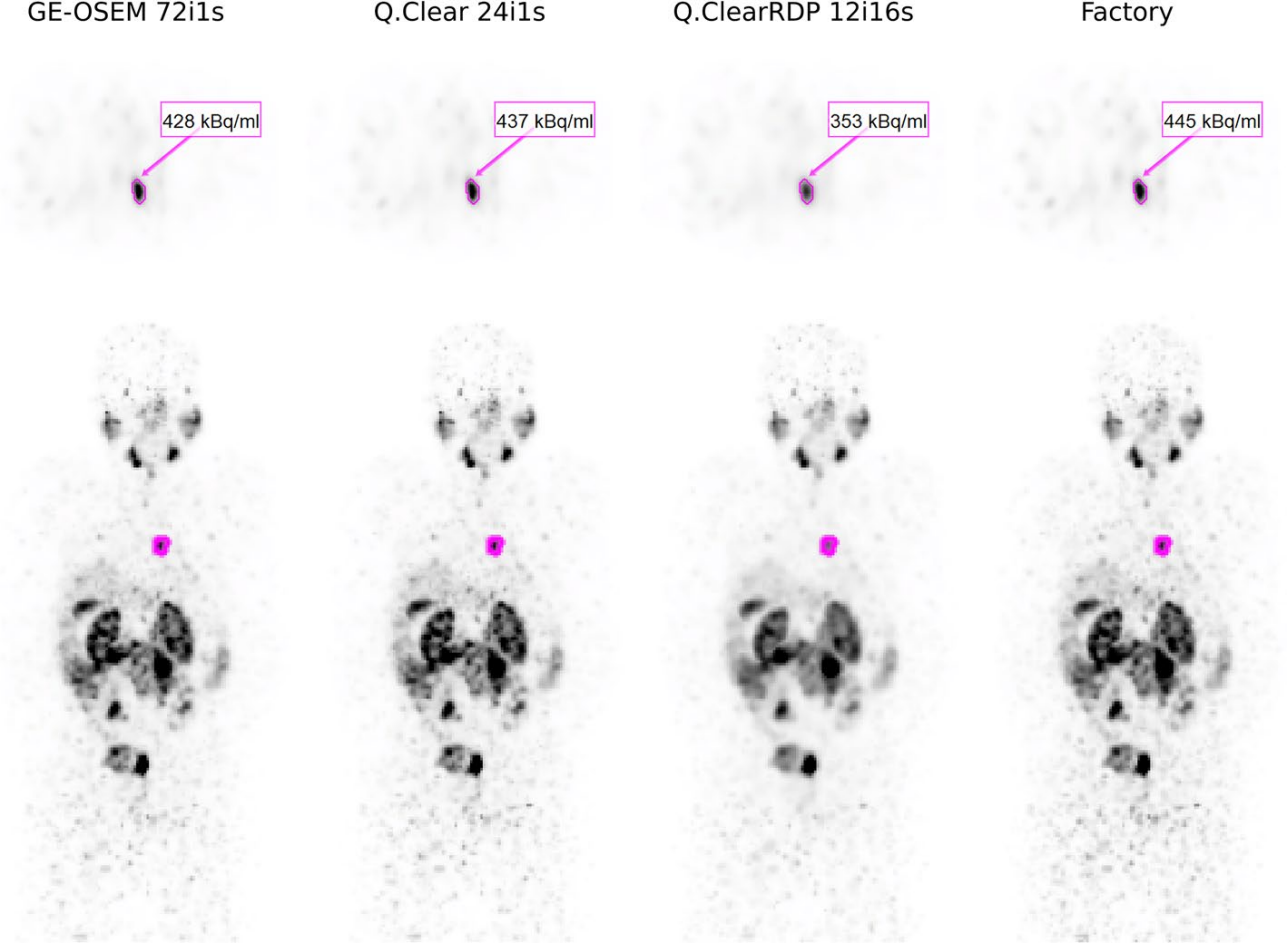
Axial view (top panel) and maximum intensity projection (bottom panel) [^177^Lu]Lu-PSMA-I&T SPECT images of a patient reconstructed with GE-OSEM 72i1s, Q.Clear 24i4s, Q.ClearRDP 12i16s and the factory protocol. The mean activity concentration of the lesions (arrow) is given for each reconstruction

To illustrate the effect of different reconstruction protocols on clinical data, an example [^177^Lu]Lu-PSMA-I&T scan reconstructed with GE-OSEM 72i1s, Q.Clear 24i4s, Q.ClearRDP 12i16s and the factory protocol is presented in Fig. 7.

## Discussion

In this study, we characterized for the first time the recently introduced StarGuide™ for quantitative ^177^Lu imaging, focusing in particular on the impact of the reconstruction protocol. For this purpose, the two reconstruction algorithms available for StarGuide™, GE-OSEM and Q.Clear (with and without RDP regularization), were compared. The final aim of this work was to pave the way to the definition of an optimal reconstruction protocol for quantitative imaging after ^177^Lu-RPT.

**Fig. 8:**
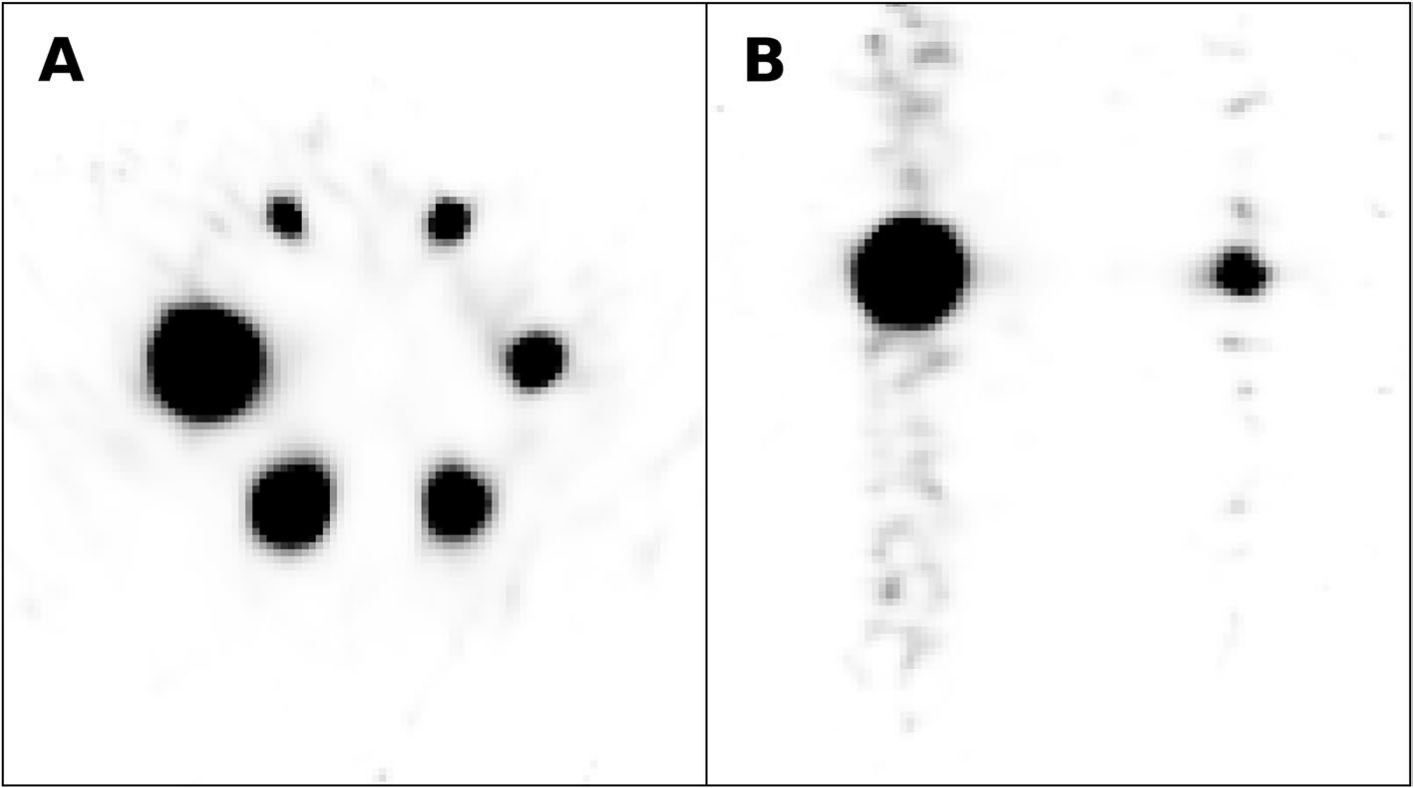
Axial (panel **A**) and coronal (panel **B**) views of the reconstruction of the NEMA phantom with cold background (GE-OSEM, 96i1s). Images were oversaturated (10% of the maximum) to show the artefact seen in the reconstruction due to the septal penetration effect

All reconstruction protocols considered in this study show a high quantification accuracy (error < 2%). Accuracy was determined by comparing the SPECT-based and the radionuclide calibrator-based activity in the externally placed enlarged VOI (VOI_outside, Table 2). The observed accuracy was found to be comparable to the results obtained by Tran-Gia et al. with a similar methodology [20]. However, independently of the reconstruction algorithm, the accuracy in the quantification of the activity concentration in the internally placed VOI (VOI_inside) was inferior, with an average underestimation of 8%, exceeding the 2% uncertainty in the ICF. Preliminary investigations—which followed the observation of the artefact shown in Fig. 8—suggested this underestimation to be due to the fact that the ICF computed according to our methodology included penetration photons that were partially excluded when considering smaller volumes such as VOI_inside. Being equipped with a fixed collimator optimized for low-/medium-energy isotopes, StarGuide™ allows more 208 keV photons to penetrate through the collimator septa than a standard medium-energy general-purpose or medium-energy low-penetration collimators.

Septal penetration possibly affected also the recovery of the activity concentration in the spherical inserts of the NEMA phantom. The RCs found in this study were lower than those reported in the literature for other cameras, where the partial volume effect was the main cause of underestimation of activity inside small volumes [22].

Further investigations are needed to assess the impact of septal penetration on the accuracy of the activity quantification in a more clinical scenario, considering different geometries and activity distributions. The possibility of reducing its impact by changing the methodology to compute ICF, for example using a VOI placed internally in the phantom, should also be explored [23, 24]. However, the fact remains that septal penetration represents a limitation of StarGuide™ with regard to the accurate determination of the activity distribution and the correct estimation of the absorbed dose to organs at risk and tumours.

For GE-OSEM, as no dependency on the number of subsets for a fixed number of updates was found, the reconstruction time can be accelerated by increasing the number of subsets without impacting the RCs and the image quality. However, the same approach is not applicable to Q.Clear and Q.ClearRDP, which use BSREM as numerical optimizer.

Compared to the GE-OSEM algorithm, Q.Clear and even further Q.ClearRDP considerably reduced the accumulation of noise for a growing number of updates (Figs. 1 and 2). The same was noticed for the Gibbs-like artefacts (Fig. 4), the appearance of which is due to the non-optional inclusion of the resolution recovery into all the considered reconstruction algorithms. Consequently, fewer updates were needed for GE-OSEM (72i1s) than for Q.Clear (24i4s) and Q.ClearRDP (12i16s) to reach similar noise levels as the factory protocol. For the proposed iteration–subset combinations, a spatial resolution comparable to that obtained with the factory protocol was determined (differences within ± 6%, Table 5). RCs for spheres larger than 17 mm were within ± 10% of those obtained with the factory protocol. For the 10 and 13 mm spheres, GE-OSEM 72i1s and Q.Clear 24i4s featured RCs more than 20% higher than those obtained with the factory protocol (Fig. 5). More evident Gibbs-like artefacts were visible for GE-OSEM 72i1s and Q.Clear 24i4s than for Q.ClearRDP 12i16s and the factory reconstructions (Fig. 6).

When looking at the reconstructions of patients treated with [^177^Lu]Lu-PSMA-I&T, no Gibbs-like artefacts were observed in lesions with volumes as large as that of the largest sphere of the NEMA phantom (> 25 mL). Visually, Q.ClearRDP 12i16s reconstructions resulted more smoothed in the abdominal area compared to the other reconstruction protocols considered (Fig. 7). Quantitatively, no statistically significant difference was observed between the tumour activity concentration measured for GE-OSEM 72i1s, Q.Clear 24i4s and the factory protocol reconstruction (Table 6). A statistically significant difference of − 8% (*P* < 0.0001) was measured when comparing Q.ClearRDP 12i16s to the factory protocol reconstruction (Table 6).

Our results prove that GE-OSEM is a valid alternative to the recommended Q.Clear reconstruction algorithm for StarGuide™, featuring comparable performances both assessed on phantom (Additional file 1: Figs. 2 and 5) and patients data. As GE-OSEM is a widely available algorithm for image reconstruction, this can come in handy when performing multi-centre studies, for which standardization of the reconstruction protocol is required [25, 26].

A major limitation of this study is the use of fixed values for the gamma and beta parameters of the RDP regularization, and the absence of the bySens tool. As first investigation of Q.Clear algorithm applied to SPECT images, we decided to limit the number of parameters and not to consider the bySens tool. For Q.ClearRDP, the choice of the gamma and beta values was therefore based on the manufacturer’s recommendations for the reconstruction of non-attenuation corrected images, for which the bySens tool cannot be enabled. Different choices of these parameters, as, for example, in the factory protocol, can have a non-negligible impact on both the image quality and the quantitative performance, especially when assessed on clinical data. In particular, preliminary investigations suggested that the use of the tool bySens has a non-negligible influence on the parameters assessed, especially when considering patients with a high body mass index.

Another limitation of our study was that we considered only single levels of activity and acquisition duration for each phantom-based analysed metric, although different results can be found for regimes of lower statistics (either obtained with a lower activity level and/or a shorter acquisition duration) often found in the clinic [19]. The response at high count rates should be also explored. Further investigations are ongoing to assess the quantitative accuracy of quantitative ^177^Lu imaging with StarGuide™ across different activity levels [27].

Finally, to assess the quantification accuracy under various attenuation and scatter conditions, further experiments using a wider range of phantom geometries are being evaluated, such as those conducted by Uribe et al. [28].

## Conclusion

To the best of our knowledge, this study is the first to investigate the performance of the newly introduced StarGuide™ system for quantitative ^177^Lu imaging, as well as the performance of the Q.Clear reconstruction algorithm for SPECT/CT, in both phantom and patients settings. Our results suggested that StarGuide™ enables accurate quantification of ^177^Lu activity, with deviations within 10% in large volumes. However, further investigations are needed to assess the impact of septal penetration on the quantitative accuracy in a more clinical scenario. When assessed on phantoms, similar values for noise (~0.15), spatial resolution (~1 cm) and RCs for spheres larger than 5 mL were found, irrespective of the reconstruction algorithm, as long as adequate reconstruction parameters were chosen. Analogue results were found in patients, provided that the bySens option for Q.ClearRDP was enabled.

### Supplementary Information

**Additional file 1**. Document containing the additional tables and figures referred to in the manuscript.

## Data Availability

SPECT and CT raw datasets are available from the corresponding author on reasonable request. All data analysed during this study are available online at https://github.com/RacheleDanieli/Quantitative-177Lu-SPECT-CT-imaging-for-personalized-dosimetry-using-a-ring-shaped-CZT-based-camera.

## Abbreviations

BSREM: Block sequential regularized expectation maximization
CV: Coefficient of variation
CZT: Cadmium–zinc–telluride
EDTA: Ethylenediaminetetraacetic acid
ICF: Image calibration factor
MRP: Median root prior
OSEM: Ordered subset expectation maximization
Q1: First quartile
Q3: Third quartile
RC: Recovery coefficient
RDP: Relative difference prior
RPT: Radiopharmaceutical therapy
VOI: Volume of interest

## References

[1] Strosberg J (2017). Phase 3 trial of 177Lu-dotatate for midgut neuroendocrine tumors. N Engl J Med.

[2] Sartor O (2021). Lutetium-177-PSMA-617 for metastatic castration-resistant prostate cancer. N Engl J Med.

[3] Danieli R, et al. Personalized dosimetry in targeted radiation therapy: a look to methods, tools and critical aspects. J Pers Med. 2022;12:205 (article number: 205. https://www.mdpi.com/2075-4426/12/2/205). 10.3390/jpm12020205 PMC8874397 35207693

[4] Strigari L (2014). The evidence base for the use of internal dosimetry in the clinical practice of molecular radiotherapy. Eur J Nucl Med Mol Imaging.

[5] Garske-Román U (2018). Prospective observational study of 177Lu-DOTA-octreotate therapy in 200 patients with advanced metastasized neuroendocrine tumours (NETs): feasibility and impact of a dosimetry-guided study protocol on outcome and toxicity. Eur J Nucl Med Mol Imaging.

[6] Del Prete M (2019). Personalized 177Lu-octreotate peptide receptor radionuclide therapy of neuroendocrine tumours: initial results from the P-PRRT trial. Eur J Nucl Med Mol Imaging.

[7] Violet J (2019). Dosimetry of (177)Lu-PSMA-617 in metastatic castration-resistant prostate cancer: correlations between pretherapeutic imaging and whole-body tumor dosimetry with treatment outcomes. J Nucl Med.

[8] Garin E (2020). Personalised versus standard dosimetry approach of selective internal radiation therapy in patients with locally advanced hepatocellular carcinoma ( DOSISPHERE-01): a randomised, multicentre, open-label phase 2 trial. Lancet Gastroenterol Hepatol.

[9] Sjögreen Gleisner K (2022). EANM dosimetry committee recommendations for dosimetry of 177Lu-labelled somatostatin-receptor- and PSMA-targeting ligands. Eur J Nucl Med Mol Imaging.

[10] Clinical Applications of SPECT/CT: New hybrid nuclear medicine imaging system. International Atomic Energy Agency; 2008.

[11] Desmonts C (2020). Evaluation of a new multipurpose whole-body CzT-based camera: comparison with a dual-head Anger camera and first clinical images. EJNMMI Phys.

[12] Song H (2023). SPECT at the speed of PET: a feasibility study of CZT-based whole-body SPECT/CT in the post 177Lu-DOTATATE and 177Lu-PSMA617 setting. Eur J Nucl Med Mol Imaging.

[13] Dewaraja YK (2012). MIRD pamphlet No. 23: quantitative SPECT for patient-specific 3-dimensional dosimetry in internal radionuclide therapy. J Nucl Med.

[14] Ahn S, Fessler JA (2003). Globally convergent image reconstruction for emission tomography using relaxed ordered subsets algorithms. IEEE Trans Med Imaging.

[15] Nuyts J, Beque D, Dupont P, Mortelmans L (2002). A concave prior penalizing relative differences for maximum-a-posteriori reconstruction in emission tomography. IEEE Trans Nucl Sci.

[16] Mustafovic, S. & Thielemans, K. Additive and multiplicative versions of the maximum a posteriori algorithm with median root prior. In: 2001 IEEE nuclear science symposium conference record (Cat. No.01CH37310), vol. 3, pp. 1783–1785 (2001).

[17] Wilk M, inventor. GE Precision Healthcare LLC, assignee. Methods and systems for scatter correction. United States Patent Application Publication 20210279917. 2021-09-09.

[18] Tran-Gia J (2021). A multicentre and multi-national evaluation of the accuracy of quantitative Lu-177 SPECT/CT imaging performed within the MRTDosimetry project. EJNMMI Phys.

[19] Marin G (2017). Accuracy and precision assessment for activity quantification in individualized dosimetry of 177Lu-DOTATATE therapy. EJNMMI Phys.

[20] Tran-Gia J, Lassmann M (2019). Characterization of noise and resolution for quantitative 177Lu SPECT/CT with xSPECT quant. J Nucl Med.

[21] Resch S (2023). Investigation of image-based lesion and kidney dosimetry protocols for 177Lu-PSMA-I&T therapy with and without a late SPECT/CT acquisition. EJNMMI Phys.

[22] Peters SMB (2019). Towards standardization of absolute SPECT/CT quantification: a multi-center and multi-vendor phantom study. EJNMMI Phys.

[23] D’Arienzo M (2016). Gamma camera calibration and validation for quantitative SPECT imaging with 177Lu. Appl Radiat Isot.

[24] Mezzenga E (2017). Quantitative accuracy of 177Lu SPECT imaging for molecular radiotherapy. PLoS One.

[25] Taprogge J, Wadsley J, Miles E, Flux GD (2021). Recommendations for multicentre clinical trials involving dosimetry for molecular radiotherapy. Clin Oncol (R Coll Radiol).

[26] Dickson JC (2023). EANM practice guideline for quantitative SPECT-CT. Eur J Nucl Med Mol Imaging.

[27] Danieli R (2023). Quantitative 177Lu SPECT/CT imaging with a ring-shaped CZT-based camera: 208 vs 113 keV photopeak. Eur J Nucl Med Mol Imaging.

[28] Uribe CF (2017). Accuracy of (177)Lu activity quantification in SPECT imaging: a phantom study. EJNMMI Phys.

