# Supplementary material for: Quantitative 177Lu SPECT/CT imaging for personalized dosimetry using a ring-shaped CZT-based camera
Source: EJNMMI Phys. 2023 Oct 18;10:64. doi: 10.1186/s40658-023-00586-z (PMC10584798; doi:10.1186/s40658-023-00586-z)
Supplement: Supplementary file 1 — Additional file 1. Document containing the additional tables and figures referred to in the manuscript. [file 40658_2023_586_MOESM1_ESM.docx]

# Additional file 1

Table 1: Image calibration factors for GE-OSEM, Q.Clear and Q.ClearRDP for 96 updates and different number of iterations and subsets.

| **Algorithm** | **Image calibration factor [cps/MBq]** | | | | |
| --- | --- | --- | --- | --- | --- |
|  | **96i1s** | **48i2s** | **24i4s** | **12i8s** | **6i16s** |
| **GE-OSEM** | 94.0 | 94.1 | 93.6 | 93.5 | 93.3 |
| **Q.Clear** | 93.9 | 93.9 | 93.8 | 93.5 | 93.3 |
| **Q.ClearRDP** | 94.0 | 94.1 | 94.0 | 93.8 | 93.5 |

Table 2: Error in the quantification of the activity in VOI_outside and activity concentration in VOI_inside for the cylinder phantom scan reconstructed with GE-OSEM, Q.Clear and Q.ClearRDP for 96 updates and different number of iterations and subsets.

| **Algorithm** | **Metric** | **96i1s** | **48i2s** | **24i4s** | **12i8s** | **6i16s** |
| --- | --- | --- | --- | --- | --- | --- |
| **GE-OSEM** | **Error VOI_outside [%]** | -1.0 | -1.0 | -0.6 | -0.6 | -1.3 |
|  | **Error VOI_inside [%]** | -9.0 | -8.5 | -8.2 | -8.2 | -8.6 |
| **Q.Clear** | **Error VOI_outside [%]** | -1.4 | -1.2 | -0.9 | -0.6 | -0.8 |
|  | **Error VOI_inside [%]** | -8.8 | -8.8 | -8.6 | -8.2 | -8.2 |
| **Q.ClearRDP** | **Error VOI_outside [%]** | -1.6 | -1.5 | -1.2 | -1.0 | -1.1 |
|  | **Error VOI_inside [%]** | -8.9 | -9.1 | -8.8 | -8.6 | -8.5 |


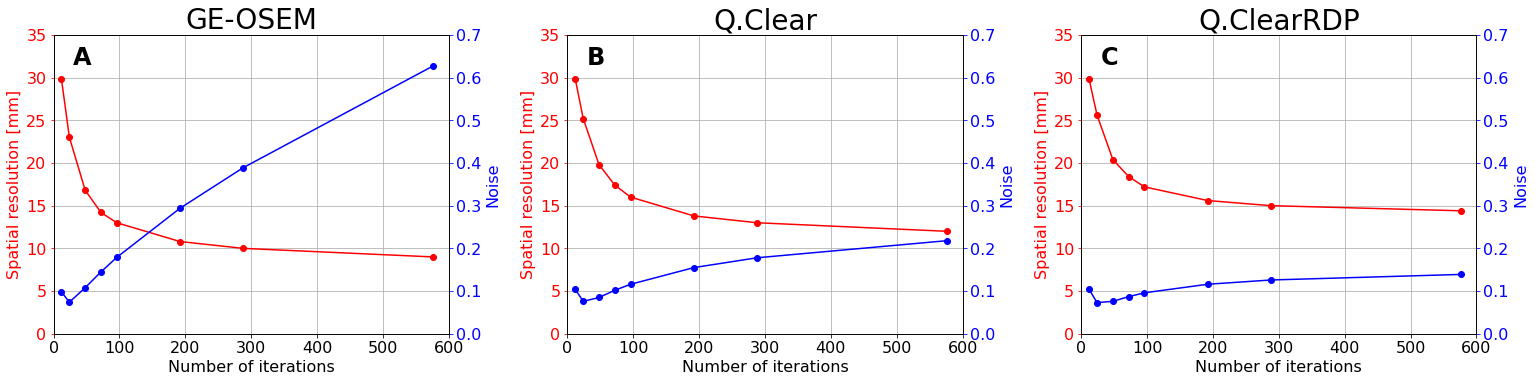


Fig. 1: Spatial resolution (red), computed on the NEMA phantom filled with hot background, and noise (blue) as function of the number of updates (1 subset) for GE-OSEM (A), Q.Clear (B) and Q.ClearRDP (C). For the factory protocol, spatial resolution and noise were found equal to 13.6 mm and 0.15, respectively.


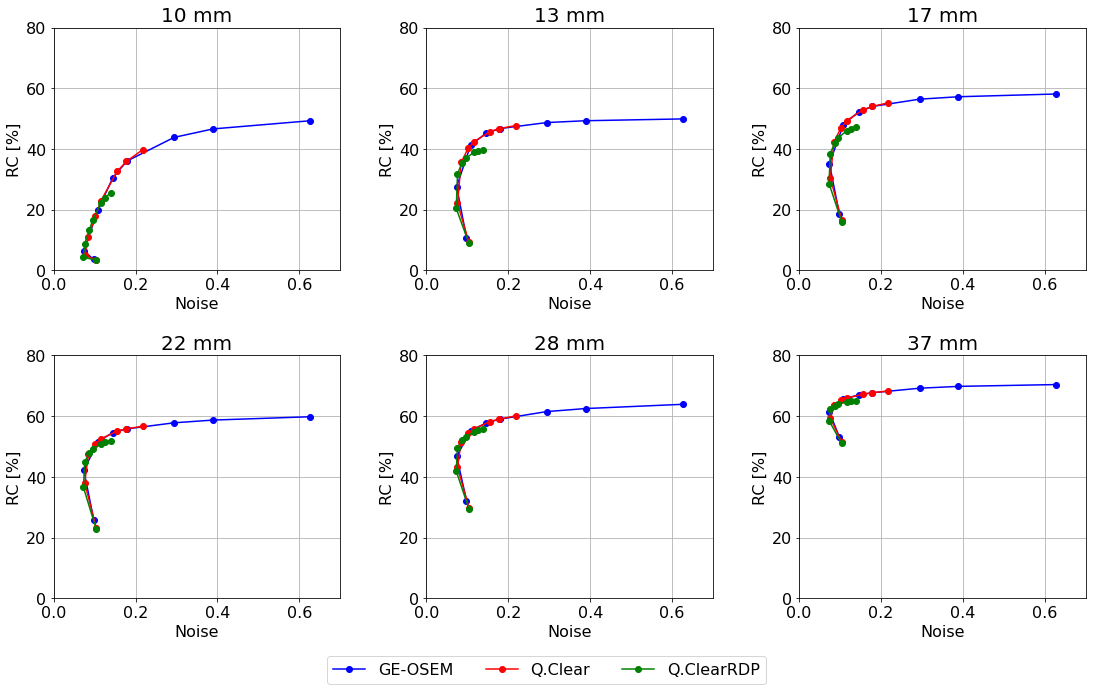


Fig. 2: Recovery Coefficients for the spherical inserts of the NEMA phantom with cold background as function of the noise for GE-OSEM, Q.Clear and Q.ClearRDP. Each data point corresponds to a specific number of iterations, namely 12, 24, 48, 72, 96, 192, 288, and 576 (1 subset), arranged from left to right.


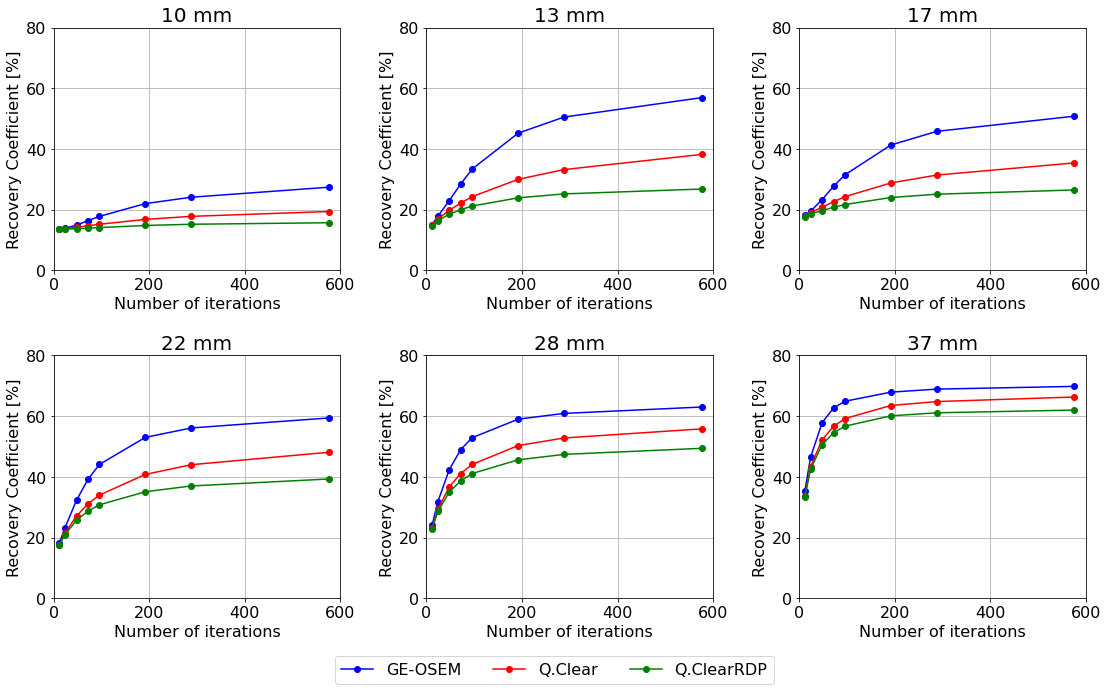


Fig. 3:Recovery Coefficients for the spherical inserts of the NEMA phantom with hot background as function of the number of iterations (1 subset) for GE-OSEM, Q.Clear and Q.ClearRDP. For the factory protocol, RCs with hot background were found equal to 16%, 29%, 30%, 44%, 53%, 63% for the 10 mm, 13 mm, 17 mm, 22 mm, 28 mm and 37 mm sphere, respectively.


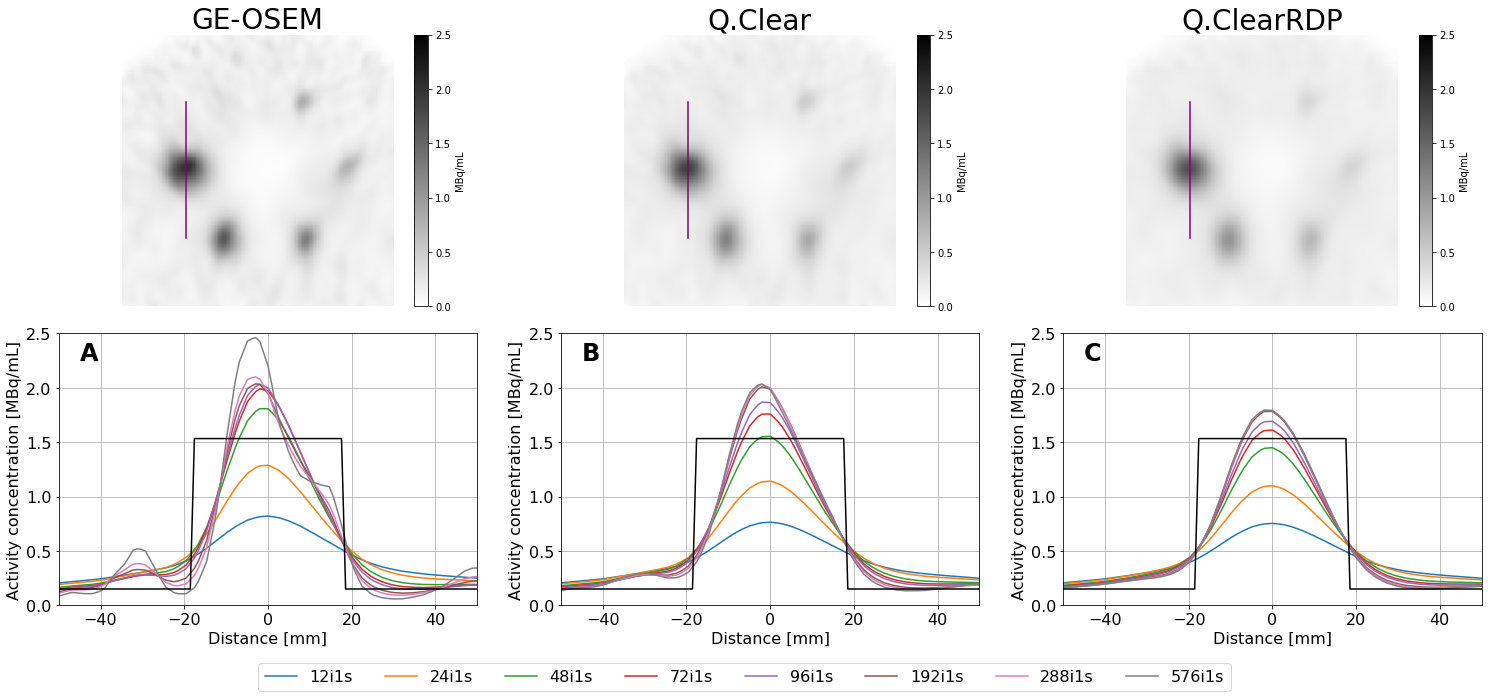


Fig. 4: Top panel - Axial views of the NEMA phantom with hot background reconstructed using GE-OSEM, Q.Clear and Q.ClearRDP (96i1s). The purple line through the biggest sphere defines the cross-section presented in the bottom panel. Bottom panel - cross-section through the largest sphere for GE-OSEM (A), Q.Clear (B) and Q.ClearRDP (C) for different number of iterations and 1 subset. The black rectangular function represents the ideal profile.


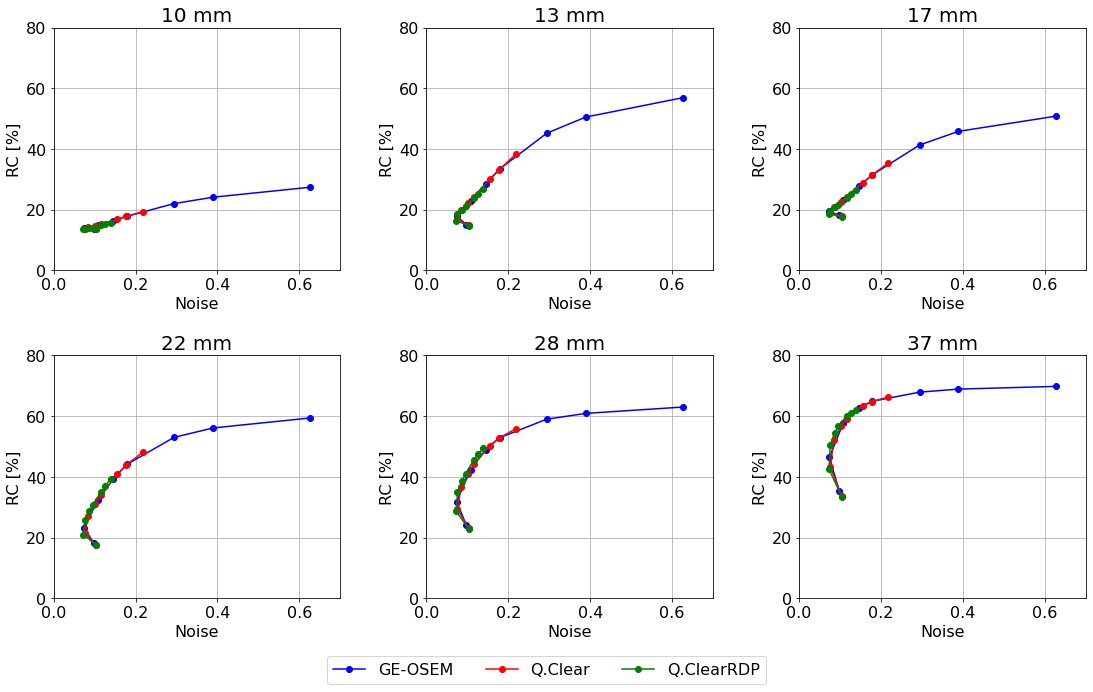


Fig. 5: Recovery Coefficients for the spherical inserts of the NEMA phantom with hot background as function of the noise for GE-OSEM, Q.Clear and Q.ClearRDP. Each data point corresponds to a specific number of iterations, namely 12, 24, 48, 72, 96, 192, 288, and 576 (1 subset), arranged from left to right.


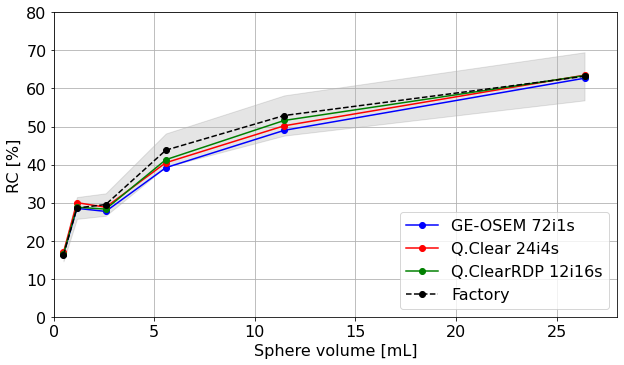


Fig. 6: Recovery Coefficients for the NEMA phantom filled with hot background reconstructed with GE-OSEM 72i1s, Q.Clear 24i4s and Q.ClearRDP 12i16s and factory protocol as a function of the sphere volume. Shaded regions correspond to ±10% the RCs computed on the factory protocol reconstruction.


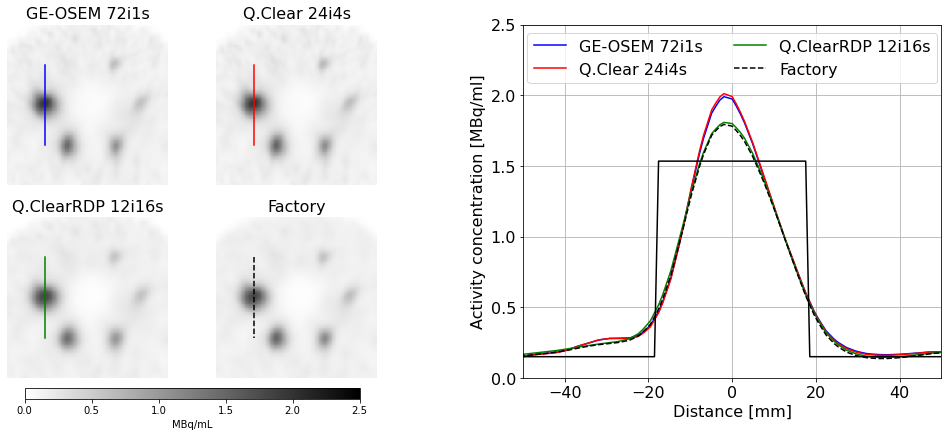


Fig. 7: Left panel- Axial views of the NEMA phantom with hot background reconstructed using GE-OSEM 72i1s, Q.Clear 24i4s and Q.ClearRDP 12i16s and factory protocol. The line through the biggest sphere defines the cross-section presented in the right panel. Right panel - cross-section through the largest sphere for GE-OSEM 72i1s, Q.Clear 24i4s and Q.ClearRDP 12i16s and factory protocol. The black rectangular function represents the ideal profile.
